# Introducing the Pearl-String Technique: A New Concept in the Treatment of Large Bone Defects

**DOI:** 10.3390/life15030414

**Published:** 2025-03-07

**Authors:** Christian Fischer, Steffen Langwald, Friederike Klauke, Philipp Kobbe, Thomas Mendel, Marc Hückstädt

**Affiliations:** 1 Department of Trauma and Reconstructive Surgery, BG Klinikum Bergmannstrost Halle gGmbH, Merseburger Straße 165, 06112 Halle (Saale), Germanymarc.hueckstaedt@bergmannstrost.de (M.H.); 2 Department of Trauma, Hand and Reconstructive Surgery, Universitätsklinikum Halle, Ernst-Grube-Straße 40, 06120 Halle (Saale), Germany

**Keywords:** Masquelet technique, non-union, diamond concept, induced membrane, bone defect

## Abstract

The reconstruction of long bone defects after the primary traumatic, secondary infectious, or tumor-related loss of substance continues to represent a surgical challenge. Distraction osteogenesis using segmental transport, vascularized bone transfer, and the induced membrane technique (IMT) are established methods of reconstruction. IMT has become increasingly popular in recent decades due to its practicability, reproducibility, and reliability. At the same time, the original technique has undergone numerous modifications. The results are correspondingly heterogeneous. This article is intended to provide an overview of the current principles and modifications of IMT, outline the causes of failure of the IMT, and introduce the pearl-string technique (PST). The PST developed in our hospital is based on the pearl-string-like arrangement of thermodisinfected, decorticated femoral heads (TDFHs) in combination with a mechanically stable osteosynthetic construct. The TDFHs are biologically activated with either an RIA or autologous iliac crest bone graft. To gain a better understanding of these variations, the surgical technique of both procedures is illustrated step-by-step in this article.

## 1. Introduction

One of the most challenging areas in musculoskeletal reconstructive surgery is the biological reconstruction of bony defects resulting from traumatic loss of substance, infections, or tumors. Smaller bone defects of up to 3 cm can usually be successfully treated relatively easily by shortening or primary cancellous bone grafting. Larger defects, on the other hand, require the use of complex reconstructive procedures.

Distraction osteogenesis via segmental transport is one of the most common techniques but is time-consuming and prone to complications [1,2,3]. The IMT surgical technique is a two-stage reconstruction procedure for bone defect treatment. In the first step, the defect is filled with a polymethyl methacrylate (PMMA) spacer and will be stabilized by osteosynthesis. As part of a synovial foreign body reaction, a membrane is induced that is characterized by good perfusion and a high concentration of growth factors, among other things. In the second step of IMT, the spacer is removed, and the defect is filled with bone substitute material while preserving the membrane [4,5,6]. The original technique of the induced membrane has been modified several times in recent years. Extensive bony defects require a large volume of bone substitutes. In this cohort, the reamer–irrigator–aspirator technique (RIA) has been established for harvesting large quantities of cancellous autograft from the medullary cavity of long bones. Bone substitute materials consisting of hydroxyapatite, tricalcium phosphate, and demineralized bone matrix (DBM) or demineralized bovine bone (DBB) were added to the graft to increase the filling volume. Bone grafts (allogeneic/xenogeneic), bioactive glasses, and calcium-based ceramics were used as supporting scaffolds. Growth factors were added to optimize osteoinductivity [5,7,8,9,10]. Despite the high biological potency of the autograft using RIA bone grafting, the results were often unsatisfactory due to sedimentation caused by gravity [11]. For this reason, custom-fit industrially manufactured 3D scaffolds were increasingly used, as they were able to prevent the sedimentation effect [12]. However, their clinical application was limited (no intraoperative flexibility, high costs, information about individualized therapy procedures, etc.). A cost-effective alternative that can intraoperatively be adapted to the defect is the use of solid allogenic donor femoral heads, which, in this form, act as a stable osteoconductive scaffold. In principle, both freeze-dried (lyophilized) or thermodisinfected femoral heads can be used. In our clinic, only thermodisinfected heads are used, as they are easier to process due to their biomechanical properties. Using these allogeneic grafts presents a valid option compared to the limited autologous grafts.

In addition to the established external fixation methods, internal osteosynthesis is also becoming increasingly important as part of initial treatment due to its high primary stability [13,14,15]. In septic reconstructive surgery, antibiotic-coated or impregnated spacers were increasingly used.

The PST is an innovative modification of the IMT based on the cornerstones of the Diamond concept. The objective in developing the PST was to emphasize the aspect of high primary stability of the construct by using internal osteosynthesis in combination with a press-fit inserted osteoconductive scaffold. The PST, therefore, conforms to the principles of primary fracture healing and presents a valid alternative to the IMT.

In our experience to date, the results of the PST are comparable to those of the IMT in terms of success and complication rates. These data are currently not subject to valid statistics. A prospective study is currently being conducted by our working group.

## 2. The IMT in the Current Literature—State of the Art

Morreli et al. and Mi et al. published the first comprehensive systematic reviews and meta-analyses on the effectiveness of IMT in 2016 and 2022, respectively [7,9]. Morelli et al. included 17 studies (n = 427 patients) in their analysis. The most common causes of segmental bone defects were post-traumatic defects (aseptic non-union, bone defects), infections (osteomyelitis, septic non-union), and tumors [9]. Following the Masquelet technique, complete osseous consolidation of the segmental defects was achieved in 90% of cases. Revision surgery was reported in 18% of cases. This was due to inadequate infection eradication and non-union. The tibia was affected in 67.2%, the fibula in 12.9%, and the femur in 19.4% of cases. The size of the osseous defects averaged 5.5 cm [9]. The interval between the two phases of IMT averaged 44 days [7,9]. Mi et al. published comparable results after using IMT after analyzing a total of 41 studies (n = 677 patients) [7]. Complete healing of osseous defects of the above-mentioned entity was achieved in 92% of cases. Persistent infections and non-union were also the most common causes of complications in this meta-analysis and led to revision surgery in 22% of cases. Tibia (59%) and femur (23%) were the most common defect localizations. The average defect length was 6.3 cm. The average time between the two phases of IMT was 76 days [7,9].

### 2.1. Antibiotic-Loaded Spacers

Morelli et al. found that the majority of PMMA spacers used during debridement (first phase) contained antibiotics (62.5%) [9]. The available literature on their use is heterogeneous. Fung et al. investigated the frequency of use of PMMA spacers containing antibiotics after analyzing 48 studies (n = 1373 patients) [16]. These were used in 69% of cases. Vancomycin and gentamicin were mainly used singularly (34%) or in combination (38%) [9]. The positive effect of the added antibiotic on infection eradication, the cytotoxic properties of some anti-infective substances, and their impact on mesenchymal stem cells and osteogenesis are still controversial. There is currently no evidence-based recommendation for the use of PMMA spacers containing antibiotics [5,6,17,18,19]. In our hospital, using medium-viscosity PMMA with gentamycin when treating cases of septic non-union or osteomyelitis has proven successful.

### 2.2. Fixation Methods

Internal, external, or combined osteosynthesis is used as part of IMT [7,9]. The choice of osteosynthesis determines the degree of stability and is crucial for primary or secondary bone healing. In our experience, the success of IMT depends largely on sufficient mechanical stability. During the reconstruction of osseous defects, the goal is to achieve intramembranous, callus-free ossification through absolute stability, similar to the principles of primary fracture healing [14,20]. In addition, a sufficiently stable graft-filling structure in the IM cavity, providing a highly osteoconductive and osteoinductive environment, is required to counteract a sedimentation effect, leading to the failure of the IMT [13,20]. Autologous bone grafts (e.g., iliac crest) are still the gold standard. However, these have their limitations in the reconstruction of larger bone defects of five centimeters or more in the lower limb or ten centimeters or more in the upper limb [21,22]. In our experience, the use of allogeneic bone grafts (e.g., solid thermodesinfected decorticated femoral heads) in combination with a high primary stability of the selected osteosynthesis has proven to be a successful approach [14,15]. The bone graft (autologous or allograft) is inserted into the osseous defect using the press-fit technique and then stabilized using internal osteosynthesis (plating or nailing). To ensure enchondral ossification of the graft, the bone surface contact between the original bone and the graft should, therefore, be less than 0.2 mm and have less than 2% strain (degree of load-dependent displacement) [6,20]. The PST combines the high primary stability of an internal osteosynthesis with the stability of the osteoconductive scaffold (TDFH) and thus respects an essential cornerstone of the Diamond concept.

### 2.3. Drawbacks and Complications

Despite the high success rates of bone healing when using the Masquelet technique for reconstructing osseous defects, it is essential to know the causes of potential failure of the induced membrane technique. Three types of IMT failure can be distinguished: septic, mechanical, and biological [13]. The persistence of infection due to inadequate debridement, failed soft tissue management, or inadequate antibiotic therapy is the most common cause of IMT failure [13,23]. Sufficient debridement is essential both in the first phase (before cement spacer interposition) and in the second phase, which represents a further opportunity to remove avascular and potentially contaminated bone [7]. Insufficient mechanical stability during the second phase is the second cause of IMT failure. A lack of mechanical stability impairs revascularization and bony incorporation of the graft, which can lead to aseptic non-union and subsequent implant failure [7,9]. Non-union after IMT usually occurs at the contact zones between the graft and the resection margins of the native bone [24]. Masquelet et al., therefore, postulated that it is crucial that the bone cement envelops the bone resection margins by two or three centimeters in the first phase and that a final decortication of the bony resection margins must take place in the second phase, taking into account the surrounding induced membrane [4,5]. The authors also described inadequate filling of the IM cavity as a further cause of mechanical failure [25]. In the area of the upper extremity, in particular, there is a risk of a sedimentation effect in the absence of stable anchoring of the graft and is considered a risk factor for the development of non-union or a refracture in the proximal part of the reconstructed bone segment [5]. The biological failure of IMT corresponds to a lack of graft revascularization despite successful infection eradication and sufficient mechanical stability. According to Mathieu et al., this form of failure is based on inadequate content or an unsuitable internal environment of the induced membrane [13].

## 3. Materials and Methods

The PST is an innovative IMT-based procedure that combines the use of RIA cancellous bone with allogeneic grafts (TDFHs). The objective of this procedure is to generate absolute stability between a stable osteoconductive scaffold (TDFH) and an internal osteosynthesis whilst taking the other cornerstones of the Diamond concept into account [17]. High primary stability, timely consolidation, and full weight bearing are comparable to primary fracture treatment, leading to high success rates with low complication rates compared to other procedures.

### 3.1. Bone Defect Reconstruction Using Solid Thermodisinfected Decorticated Femoral Heads and RIA Cancellous Bone

Bone defects of up to 5 cm in the lower limb and up to 10 cm in the upper limb can usually be completely reconstructed with autologous bone. For this purpose, bi- or tricortical iliac crest grafts are used, which are inserted into the bone defect using the press-fit technique.

**Figure 1:**
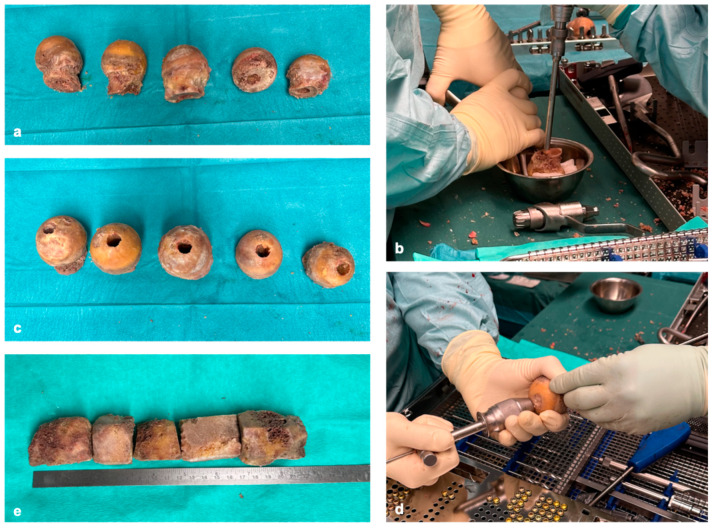
Thermodesinfected femoral heads (**a**), cannulation of the femoral heads (**b**), cannulated femoral heads (**c**), reaming the femoral heads 1 mm over the nail diameter (**d**), femoral heads after removing the cortical bone (**e**).

Reconstruction of extensive dia-/metaphyseal defects of more than 10 cm requires using solid grafts. Based on our experience, these critical defect sizes cannot be reconstructed with autologous grafts alone (e.g., iliac crest grafts). A combination of autologous and allogenic grafts is therefore required. In our clinic, TDFHs are used as allogenic grafts because they have a high mechanical stability as an osteoconductive matrix (Figure 1a,c,e).

TDFHs are commercially available in unlimited quantities and are significantly cheaper than synthetically produced scaffolds. The number of donor femoral heads used depends on the extent of the segment defect. Depending on whether parts of the femoral neck were removed or preserved when the femoral heads were resected, cancellous cylinders between 3 and 6 cm can be created (Figure 1e). This means that two to three femoral heads are required for a 10 cm defect. In case osteosynthesis is performed with an intramedullary nail, the femoral heads are cannulated and drilled 1 mm larger than the nail diameter (Figure 1b,d). The cortical bone is completely removed, as, in our experience, revitalization of the donor’s bone with ingrowth of blood vessels only occurs effectively if the donor’s bone has a cancellous surface (Figure 1a–e).

**Figure 2:**
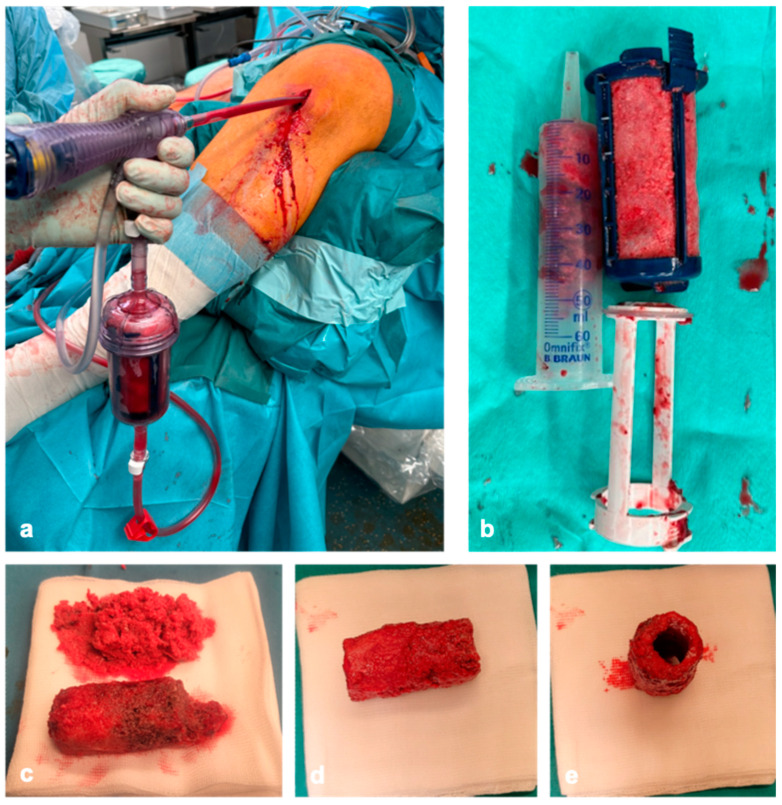
Harvested cancellous bone with the reamer–irrigator–aspirator system (Synthes, West Chester, Pennsylvania, USA) (**a**,**b**), coating the bone cylinders with RIA cancellous bone ex situ (**c**–**e**).

As the volume of cancellous bone removed from the iliac crest is limited, cancellous bone is harvested using RIA in the reconstruction of larger bone defects. If the segmental defect is on the tibia, the cancellous bone is harvested retrograde from the equilateral femur using the RIA. In case implants are present on the proximal femur, we harvest the cancellous bone retrograde. Alternatively, the contralateral femur or tibia can be used as a harvesting site. To avoid iatrogenic fractures during harvesting, a correct entry point, measurement of the medullary canal, and intraoperative imaging in two planes are required. In combination with donor bone, 10 cm^3^ of cancellous bone is sufficient for the reconstruction of a segmental defect of 4 cm. Accordingly, around 50 mL of RIA cancellous bone is required for the reconstruction of a 20 cm long defect (Figure 2a,b).

**Figure 3:**
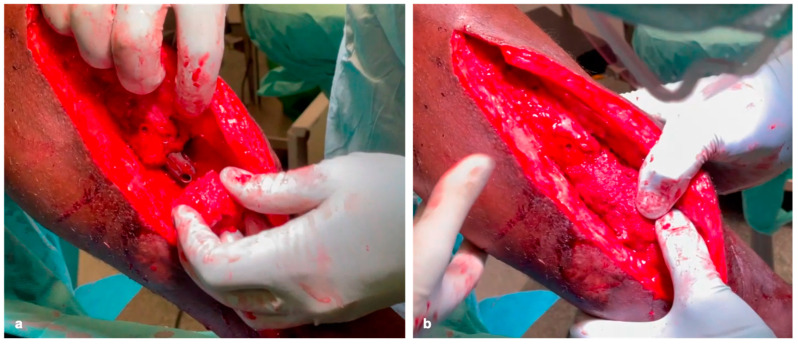
Threading the TDFHs onto a tibial nail (**a**), press-fit insertion of the TDFHs, and advancing of the nail distally (**b**).

The heads are then adjusted to the diameter of the defect (Figure 1d and Figure 2e). After coating the bone cylinders with the RIA cancellous bone (Figure 2c–e) ex situ, they are then inserted into the defect like a string of pearls using the press-fit technique and stabilized with plates and/or nails to generate high primary stability (Figure 3a,b).

Sufficient RIA cancellous bone must be attached to the contact surfaces of the femoral heads and the resection edges of the original bone (Figure 3b). If the insertion of the bone grafts using the press-fit technique is only partially successful, compression can be applied via the implant or externally. Smaller defects are then filled with the remaining RIA (Figure 2c).

If intramedullary nail osteosynthesis has already been performed in a previous procedure, the femoral heads are cut in half and attached around the nail. They can be secured by using a cerclage or an additive locking plate. If osteosynthesis is performed with locking plates, the heads are inserted en bloc. Fixation is then performed with head-locking screws.

**Figure 4:**
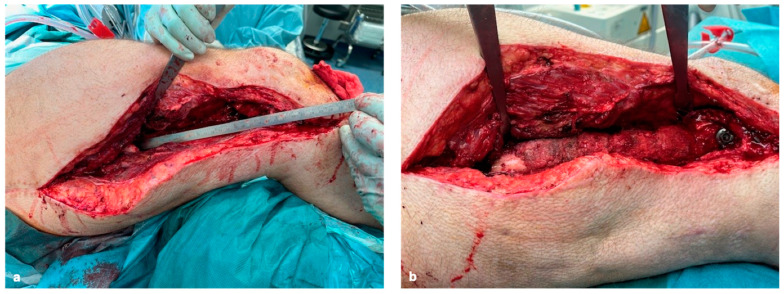
Femoral bone defect measuring 19 cm (**a**), reconstruction of the femoral bone defect using 5 cannulated, RIA-coated, solid TDFHs using the pearl-string technique (**b**).

**Figure 5:**
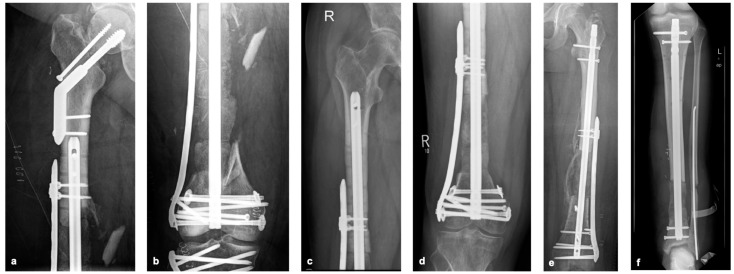
Postoperative X-ray after reconstruction of bone defects of the femur and tibia using the pearl-string technique (PST), case from Figure 4a,b 48 hours after surgery (**a**,**b**), other cases (**c**–**f**) describing the PST in other patients at 3-month follow-up.

Based on our experience, the absolute stability of the selected osteosynthesis and bone graft is essential in the reconstruction of (larger) bone defects. If defects are present in the isthmus area of long bones, osteosynthesis with a sufficiently strong nail may be sufficient. If there is insufficient stability, locking plates should be used additionally (Figure 4a,b). In the joint area, additional locking double-plate osteosynthesis is typically preferred in our hands (Figure 5a–f).

### 3.2. Aftercare

**Figure 6:**
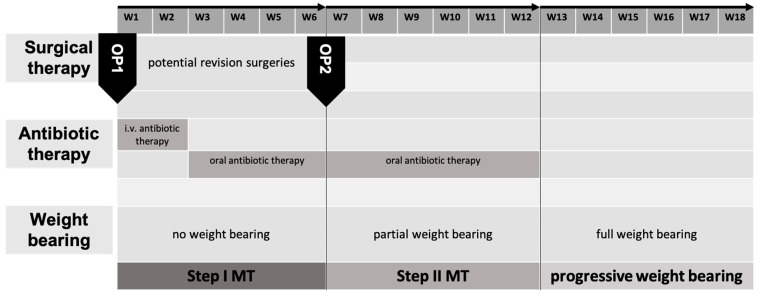
General aftercare concept of the induced membrane technique (IMT) and the pearl-string technique (PST) in our hospital.

The described modifications of the IMT combine stable internal osteosyntheses with stable cancellous bone grafts. This allows, at a minimum, postoperative exercise stability in all cases. Regarding defects of the lower extremities, applying a load (partial weight-bearing) of 20 kg is generally possible immediately postoperatively. In contrast to other methods of osseous defect reconstruction, this minimizes long-term functional impairments. After 6 weeks, weight bearing is gradually increased, and full weight bearing should be achieved 12 weeks postoperatively. Deviations from the standard may be indicated depending on the radiological checks or individual factors such as the ability to bear partial weight or body weight. We typically carry out radiological follow-ups after 6 and 12 weeks and after 6 and 12 months (Figure 6). In addition to assessing the progress of consolidation, it is important to recognize possible implant complications at an early stage.

Overall, it can be stated that the PST allows for early weight bearing compared to other procedures. The axial load transfer from the proximal to the distal fragment via the intramedullary implant and the press-fit insertion of the stable scaffold (TDFH) enable this. The high primary stability/absolute stability of the construct achieved this way is comparable to the principles of primary fracture healing.

## 4. Discussion and Conclusions

In our experience, in addition to the gold standard of using autologous bone grafts, using allogeneic bone grafts for reconstructing larger bone defects has proven successful. Achieving absolute stability between the original bone and the bone graft is essential. If possible, the bone graft should be inserted using the press-fit technique. Additional compression can be applied using standard internal, external, or combined osteosynthesis. When using intramedullary nails, additive locking plates can be used if necessary. In regions close to the joints, double-locking plate osteosynthesis is used.

However, mechanical stability is only one cornerstone to treat bone defects. Bone defects caused by infection require adequate treatment through radical surgical debridement combined with pathogen-specific antibiotic therapy. Last but not least, the other essential principles of the Diamond concept must be applied in order to achieve a successful biological bone reconstruction.

To this day, 41 patients have been successfully treated in our hospital using the pearl-string technique. The average bone defect was 9.7 cm long (range: 5–22 cm) and 3.4 cm wide (range 2–6.5 cm). In three cases (7.3%), the IMT had to be completely or partially removed due to persistent infection. After treating the infection, the IMT was successfully performed again in these patients. In all other cases (92.7%), primary consolidation was achieved without the need for revision. In our experience, the length of the bone defect has no significant influence on the consolidation rate or the time until full weight bearing is achieved.

As this article is a casuistic description of a new surgical procedure, there are some limitations to this study. None of the data regarding consolidation and complication rates are currently subject to valid statistics. Future publications by our research group will address this aspect.

The results of the PST are promising, considering the results of two meta-analyses of the original IMT [7,9]. Once the data from the current prospective study on the PST have been analyzed, it will be possible to make statistically valid conclusions about consolidation and complication rates in comparison with IMT. In conclusion, it can be stated that the pearl-string technique, when performed correctly, has proven to be a successful treatment option when treating large bone defects.

## Data Availability

The data presented in this study are available on request from the corresponding author due to (specify the reason for the restriction).

## Abbreviations

The following abbreviations are used in this manuscript:

IMT	Induced Membrane Technique
PST	Pearl-String Technique
TDFH	Thermodisinfected Decorticated Femoral Head
PMMA	Polymethyl Methacrylate
RIA	Reamer–Irrigator–Aspirator
DMB	Demineralized Bone Matrix
DBB	Demineralized Bovine Bone
